# Slack Is Needed to Solve the Shortage of Nurses

**DOI:** 10.3390/healthcare12020220

**Published:** 2024-01-16

**Authors:** Frits Van Merode, Wim Groot, Melline Somers

**Affiliations:** 1 Care and Public Health Research Institute, Maastricht University, 6200 MD Maastricht, The Netherlands; 2 Maastricht University Medical Centre+, P. Debyelaan 25, 6229 HX Maastricht, The Netherlands; 3 Maastricht Graduate School of Governance, Maastricht University, Boschstraat 24, 6211 AX Maastricht, The Netherlands; w.groot@maastrichtuniversity.nl; 4 Research Centre for Education and the Labour Market, Maastricht University, Tongersestraat 49, 6211 LM Maastricht, The Netherlands; melline.somers@maastrichtuniversity.nl

**Keywords:** healthcare, workforce, shortages, queuing theory, information theory, coordination, slack, job design, task design, complexity theory, complex system

## Abstract

Healthcare systems are facing a shortage of nurses. This article identifies some of the major causes of this and the issues that need to be solved. We take a perspective derived from queuing theory: the patient–nurse relationship is characterized by a scarcity of time and resources, requiring comprehensive coordination at all levels. For coordination, we take an information-theoretic perspective. Using both perspectives, we analyze the nature of healthcare services and show that ensuring slack, meaning a less than exhaustive use of human resources, is a sine qua non to having a good, functioning healthcare system. We analyze what coordination efforts are needed to manage relatively simple office hours, wards, and home care. Next, we address the level of care where providers cannot themselves prevent the complexity of organization that possibly damages care tasks and job quality. A lack of job quality may result in nurses leaving the profession. Job quality, in this context, depends on the ability of nurses to coordinate their activities. This requires slack resources. The availability of slack that is efficient depends on a stable inflow and retention rate of nurses. The healthcare system as a whole should ensure that the required nurse workforce will be able to coordinate and execute their tasks. Above that, workforce policies need more stability.

## 1. Introduction

Around the world, healthcare systems are experiencing shortages of healthcare workers, particularly nurses [1,2,3]. While labor shortages and an oversupply of workers have alternated in the past, a wide range of countries are currently facing a structural shortage of qualified nursing staff. These structural shortages cannot be solved completely by more investments in training and attracting staff; they also require healthcare provision to be restricted, and the organization of care delivery needs to be changed. An example is relying more on informal care by family members rather than professional care givers.

In aging societies, healthcare workers increasingly leave the labor market due to retirement and gradually transition from healthcare providers to healthcare users, while the inflow of younger cohorts is insufficient to meet the increasing demand for healthcare workers. On top of that, the healthcare sector is frequently incapable of competing with the wages and working conditions offered in other industries. A higher retention rate of healthcare workers in the profession could mitigate this shortage. In this article, we discuss the working conditions required for a healthy retention rate of registered or licensed nurses: nurses need slack time to be able to perform their tasks well and to coordinate the activities for which they are responsible. We argue that slack time is essential for retaining nurses in the healthcare sector.

In this article, we focus mostly but not exclusively on nurses. With a global workforce exceeding 28 million, nurses constitute the largest group of healthcare professionals [1]. The International Council of Nurses (ICN) estimates that 13 million additional nurses will be required to address existing workforce shortages [2]. According to the WHO report ‘Health and care workforce in Europe: time to act’ [3], European countries are at a critical juncture, requiring “strategic planning and smart investments”. Given this shortage of 13 million nurses, this is true for other regions as well.

Workforce availability strongly relies on long-term forecasts and planning. In this study, we show how instability in workforce supply may continue despite workforce planning and forecasts. The time horizon plays a significant role here. Nurse shortages have been expected and forecasted in most countries for many years, but there have also been periods in which nurses have left the healthcare sector because of a lack of jobs. The current shortage of nurses in European and North American countries is not solely attributable to demographic transitions such as the aging of the population. To a large extent, it is also a consequence of and a response to the short-term austerity measures implemented in the aftermath of the 2008 global financial crisis [4,5,6]. The COVID-19 crisis has largely exacerbated this workforce problem. While the need for coordination by nurses is not new, the current staff shortages put pressure on the available slack time for workers and push the boundaries of coordination possibilities to a critical juncture. The short- and medium-term nurse shortages of the past have evolved into a structural problem, resulting in a continuous shortage of nurses with limited possibilities for substantial training expansion.

In this study, we approach healthcare as a system and focus on slack in the system as a condition for the system to function well. We define slack as the amount of time that a task can be delayed without affecting other tasks or the completion of the task itself. This definition aligns with the theory of Ashby: if a system, regardless of its nature, is confronted with uncertainty, there is no assurance that it will have enough requisite variety to effectively address the uncertainty without incorporating redundant capacity [7,8]. We try to identify this redundancy and the lack or excess of slack in the different layers of the healthcare system and demonstrate how the lack of slack creates poor job conditions for nurses and negatively influences their retention. We take an analytical approach grounded in queuing theory. The following layers of the healthcare system will be analyzed:

1. The patient–healthcare professional relationship.
2. The single queue–single server model: the office hours at outpatient clinics and the work completed by nurses in the ward as part of their shift.
3. Pooled queues and/or servers organized in parallel or in sequence.
4. Staff rosters (a)synchronized with production schedules.
5. Healthcare systems with blocking.

We argue that a lack of slack and a lack of control both have a negative impact on job quality. We present theories and evidence from the literature to make a connection between the work system in healthcare and poor job characteristics. Our analyses focus on the way that nurses’ work is organized and the effects this has on (1) the well-being of nurses, (2) how nurses like their job, and (3) the negative job conditions leading nurses to fall ill and/or leaving their job. Hence, this paper tries to explain why nurses are ‘pushed out’ (‘push factors’) of their job by their working conditions. We do not discuss the ‘pull factors’ that explain why jobs in other economic sectors are more attractive and prompt nurses to quit their job because of better prospects elsewhere. Concerns about the working conditions of nurses are indeed prevalent. For instance, a study conducted by the authors of [9], encompassing nurses from 12 European countries, revealed widespread dissatisfaction with working conditions. Common issues included the rationing of care due to high workloads, with nurses expressing concerns about the overall quality of care and a perceived lack of attention from management. Additionally, a prevalent intention among nurses to leave their jobs was observed.

Although much of our discussion is about nurses and doctors, the emphasis is on nurses. There are many similarities between these two professional groups, but when considering the push factors, there are some important differences. First, the autonomy and bargaining power of doctors is much larger. Doctors also have more options to foster their interests outside of the healthcare sector: they have strong and powerful professional associations and generally have good access to media and politics and possibilities to bypass hospital management if needed [10]. Second, nurses’ basis of power is mostly restricted to and depends on access to the management of the healthcare provider, and nurses often have limited access to top-level management. According to a study conducted in a large teaching hospital in the Netherlands, the “perceived subservient position of nurses” can be considered as the main root cause of decreased nurse staffing levels [11]. Of course, there are exceptions, such as Magnet^®^ organizations [12].

Healthcare, as a system, has to deal with uncertainty [13]. To analyze uncertainty, we use queuing theory, wherein models describe customers requiring services offered by suppliers. A summary of queuing theory is provided in Appendix A. These services are defined as a given collection of tasks [14]. In queuing theory, the customers can be considered to be the patients and the staff waiting for patients or for each other. Healthcare organizations can be considered as queuing networks, as many of the tasks that have to be performed can pooled and/or connected through patient and staff flows. Patient and staff flows may lead to queuing. Depending on the pooling of queues and structuring of tasks into processes, performance may differ even with the same customer arrival rates and task times. In this study, we analyze healthcare as a system of queuing processes. A queuing process is defined by John Little, one of the main founders of queuing theory, as “a mathematically specified operation in which units arrive, wait, and then leave” [15]. 

From this perspective, we analyze healthcare as a complex system with high coordination (synchronization) needs between services. If the design of this system and/or its coordination are not sufficient, patients will have to wait, and staff will simultaneously be overburdened and underutilized. The purpose of this article is to study and demonstrate how queuing theory and an information-theoretic interpretation of coordination can assist in identifying the causes of nurse shortages and finding solutions for them.

## 2. Slack in Operations Systems

### 2.1. Simple Queuing Systems

**Figure 1:**
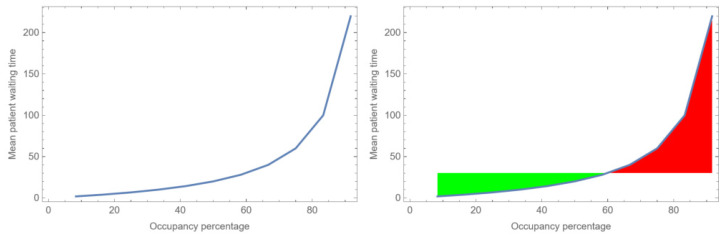
The relationship between the occupancy rate of nurses or doctors and waiting times for patients in a simple office hour system when arrivals and process times are uncertain. The x-axis indicates the occupancy rate, while the y-axis depicts the mean waiting time in minutes. The green area shows the acceptable mean waiting times for patients. The red area depicts unacceptable mean waiting times for patients.

Systems without slack (buffers) do not exist. If slack is not built into the system, the system will be out of control. For example, suppose we have a nurse or a doctor in a hospital who is scheduled to work from 8.00 a.m. to 12.00 p.m. Every half an hour, a patient is scheduled for a visit with a mean time of 20 min (exponentially distributed) (see Figure 1 below). Does this work, and what is the chance that the doctor or nurse will still see patients after the scheduled time has ended? Moreover, how much waiting time is there? In this example, the scheduled slack in this system for the nurse or the doctor is 20 min per hour, i.e., 33%. However, as office hours often exceed 12:00 a.m., the real slack is typically larger. The real slack depends on the exact probability distribution of process and arrival times. When the real office hour period exceeds the scheduled time, the occupancy rate is often considered to be above 100 percent. For example, an office shift with scheduled patient visits between 8.00 a.m. and 12.00 a.m. and with a nurse or doctor waiting for patients for 30 min but with an excess time of 60 min is often considered a very busy office shift with an occupancy rate of

$$\frac{1\;p.m.-8\;a.m.-30\;min}{12\;p.m.-8\;a.m.}=112.5\%$$

However, the actual occupancy rate of capacity is as follows:

$$\frac{1\;p.m.-8\;a.m.-30\;min}{1\;p.m.-8\;a.m.}=90\%$$

Due to the uncontrolled extension of the office hours, the denominator of the occupancy degree calculation also increases, causing a seemingly more manageable workload. At the same time, this extension could cause problems for doctors, nurses, and patients who have scheduled appointments. Other activities might have been planned between 12:00 a.m. and 1:00 p.m., and these activities therefore become disturbed. The rearrangement of schedules and activities not only increases the workload but also increases the required coordination. Coordination efforts may go unnoticed when only looking at the primary tasks of nurses and doctors. Moreover, by not designing buffers, systems create their own buffers in an unintended and uncontrolled way. This can also be referred to as open-loop system behavior, which we will discuss below in more detail. A description of open-loop and closed-loop systems in relation to queuing theory is provided in Appendix A. Obviously, some measured could be taken in this example. For example, patients could come earlier than their scheduled time, filling the waiting room. This is common practice in healthcare facilities, but it implies that patients are used as buffers. Alternatively, one could schedule more patients than the number of patients that fit in the scheduled office hours. The goal would be to compensate for the ‘lost time’ when patients do not show up. ‘Overbooking’ adds to the variability of the length of office hours and uses nurses/doctors and patients as buffers, assuming they have slack time. 

Figure 1 presents an example of a simple queuing system where individual encounters are assigned to office hours of specific doctors or nurses. The probability that an occupancy rate of 100% can be planned is very low. Using waiting time as an indicator for system stability reveals an exponential decrease in the system’s relative stability, and thus controllability, with an increase in occupancy. Suppose that a certain mean waiting time for patients is considered acceptable. In the right curve, two areas can be identified. The green area shows the acceptable mean waiting times. The red area depicts unacceptable mean waiting times. With the increase in occupancy, the mean waiting time exponentially increases. Due to variation in arrival times and the time at which the doctor or nurse sees the patient, achieving complete control is nearly impossible. The control that is required when a patient arrives focusses on managing the time for that specific patient within certain limits, considering that another patient will soon occupy the waiting room. In such a queuing system, there exists both a high and low utilization of staff, even if, on average, there is sufficient capacity. Consequently, the system requires a built-in degree of underutilization or slack. The commonly sought-after ‘rule of thumb’ or ‘golden standard’ is an occupancy rate of 0.85, especially in hospital wards. However, an occupancy rate of 0.85 can be too low but also too high, depending on the context [16]. For more complicated queuing systems or for systems of queuing systems (e.g., care chains, the outpatient clinic as a whole, etc.), a rate of 0.85 is deemed too high and can result in ‘blocking’, a concept we will discuss below in more detail.

**Figure 2:**
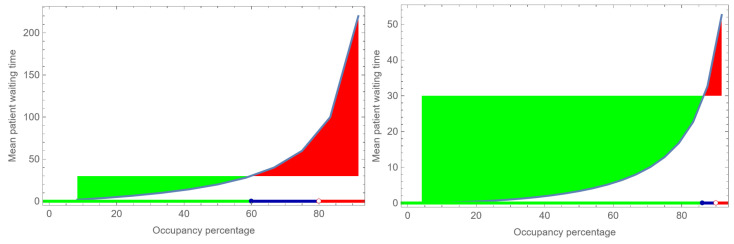
The effect of pooling the queuing patients and staff of a one-server system to a two server system. Three states are distinguished: low-coordination need (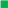), high-coordination need (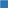), and chaos (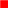).

Figure 2 shows the relation between the occupancy rate and the mean waiting time for patients in a scenario where both arrivals and visit times at the doctor or nurse are exponentially distributed. In this distribution, most waiting times cluster around and slightly below the average, but some instances are significantly longer. Given a known distribution of waiting times and set maximum acceptable waiting times, we can determine the occupancy rate and the slack staff time. Additionally, we could also include norms for slack and coordination time and derive the occupancy rate. These calculations do not need to have the same occupancy rate as an outcome. The curve below the green area indicates the occupancy rate with acceptable mean waiting times. The curve above the red area depicts the unacceptable mean waiting times. With an increase in occupancy, the coordination effort to keep waiting times and visit times in control increases, and after a certain point, coordination becomes ineffective, a concept referred to by Seeley as ‘Murphy’s point’ [17], (p. 80), as it brings the system into chaos. To ensure the stability of the system, the maximum acceptable waiting time should be below Murphy’s point. The distance between the occupancy with the maximum mean waiting time and Murphy’s point is an area with a heavy coordination effort. Suppose Murphy’s point is at an occupancy level of 80% (where the average waiting time is 85 min). In that case, the heavy coordination zone lies approximately between occupancy levels 60% and 80%. This is depicted as the blue zone in Figure 2. It is worth considering that the increase in coordination may start well before reaching the 60% occupancy threshold.

To increase occupancy levels without increasing waiting times, one can consider pooling staff and patients. Figure 2 demonstrates the effect of pooling the same type of capacity while simultaneously combining the same type of healthcare demand. The concept of pooling is explained in more detail in Appendix A. For example, one could combine two office hours of two doctors and nurses with the same processes and with one joined queue. The variation in inter-arrival and process times are the same; only the total volume doubles. The effect is that waiting times decrease substantially (see the right panel of Figure 2). The area of chaos and that of heavy coordination load reduce enormously and shift to higher occupancy levels. The effect is also that the need for slack decreases. However, reducing the area of heavy coordination load also requires caution, as there is a chance that, at any point in this area, with a small increase in the demand or processing time, the likelihood of entering into the chaos area increases. This means that an increase in productivity requires a high degree of stability, which can only be achieved by a closed-loop system. 

Staff and patient pooling are very effective in making working times more predictable, preventing overtime, creating a better work environment, and reducing the need for safety capacity (and thus redundancy) [18]. An obstacle to implementing the pooling strategy is the inherent challenge of preventing an increase in the heterogeneity of the case mix [18]. If the case mix becomes too heterogeneous, the effects of pooling may become negative, as more redundancy and/or investment in extra coordination is required. The difficulty is that it might take a considerable amount of time to observe, analyze, and redevelop a system that has been designed for a homogenous patient population but instead serves a more heterogeneous population. Case mix changes influencing the efficiency and quality can be diverse: a heterogeneous case mix may become more homogeneous in the future. Alternatively, a homogenous population gradually requires higher workloads when it becomes more heterogeneous. Staff and patient pooling can also change workforce requirements, either gradually (the population at which the system is directed changes) or suddenly (the system is redesigned to adapt to the changed patient population). Pooling may require the centralization or concentration of health services, often at the regional level, which comes with both positive and negative implications. One drawback of centralization is the potential reluctance of nurses to travel longer distances.

In summary, a deficiency in slack is likely to amplify waiting times for patients, doctors, and nurses, even if there is, on average, sufficient capacity available. This lack of slack reduces opportunities for staff to coordinate activities to match supply and demand. Consequently, nurses and doctors not only perceive a loss of control but also experience diminished autonomy. This, in turn, leads to increased work pressure, moral distress, and stress, contributing to overall dissatisfaction with job conditions and potential illness. Ultimately, the absence of slack adversely affects retention and increases the likelihood that nurses will drop out due to sickness or quit their job. This creates a vicious circle, further diminishing the remaining nurses’ slack time and exacerbating the shortage of nursing staff.

### 2.2. Systems of Tasks: Parallelization

Pooling two office hours with identical demand and supply characteristics not only reduces uncertainty but also minimizes the necessary coordination effort. However, for efficient capacity utilization, there should be a sufficient number of patients (1) and staff (2) to pool. Moreover, there are time restrictions: patients have to be seen within a certain time window (3). 

Care organizations often choose to split office hours to create more homogenous patient populations for each office hour. This is a good pooling strategy if there is a sufficient number of patients for these specialized office hours. This strategy implies that the tasks to be performed are more standardized. Dividing office hours in this manner, served by different staff, leads to the emergence of task shifting. In their review of task shifting and skill mix changes, Meda et al. [19] define task shifting as the expansion of roles within a certain category of healthcare professionals, the interchange of tasks between them, or the creation of new professions through innovations (e.g., as a result of new technology) requiring new skills. Specialized office hours cause the doctors and nurses involved to become more specialized in certain categories of patients and tasks. Under certain conditions, this is beneficial for the quality of care, efficiency, and coordination efforts. When specialized office hours are scheduled, patients are ‘sorted’ into specific office hours, and thus towards specific doctors or nurses. This reduces the task uncertainty of nurses and doctors and will reduce both the variation in utilization at the level of the office hour as in the workload. The same reasoning applies to wards: specialized wards will have more standardized tasks and thus less variation given a certain occupancy level. In principle, the processing rate, and thus the occupancy rate, in both in office hours and wards may increase substantially when they become more specialized. Task specialization can typically be observed in academic medical centers and big hospitals, as well as in specialized clinics. Apart from the discussion of whether there is some optimal point between specialists and generalists, the effectiveness of the splitting and pooling strategy remains unclear from a coordination and efficiency point of view. To determine whether patients and staff should be pooled, the following considerations need to be made:

(1) To be able to ‘fill’ the specialized office hours and wards, there should be ‘enough’ patients (e.g., by concentrating certain medical services in the region). Therefore, splitting and pooling can and sometimes need to go together.
(2) To ensure the continuity of office hours or ward operations for an extended duration, it is essential for the demand to remain stable. In situations where demand fluctuates, staff flexibility becomes crucial, allowing staff to be assigned to different office hours or wards. This flexibility is particularly important for nurses who may be required to attend to wards with high workloads or staff shortages.
(3) The sorting of patients should be effective.

If the sorting process of patients is not effective, the system will not work, as heterogeneous patients occupy office hours that are meant for homogeneous groups of patients. This sorting also needs capacity, which should be accounted for when evaluating the total staff assigned to patient care. What is being created when organizing these sets of parallel office hours, wards, tasks and jobs is in fact a large, complex system that requires substantial coordination. In a situation where the volume of demand and its case mix are constant and where it is possible to match with specialized capacity, this would be good idea. However, in reality, the above conditions are often not fulfilled, which leads to low occupancy rates for certain office hours and (too) high rates for others, as well as long access and waiting times for patients. Low and high utilization may also change over the subsequent months and years. Therefore, the management of specialized office hours requires a delicate balance of standardization and flexibility. This involves the short-term deployment of staff across various patient types and tasks, adjusting the office hours or work division scheme for nurses and, in the long run, providing (re-)training opportunities for nurses. The latter will become increasingly difficult as nurses become more specialized, possibly restricting their flexibility or willingness to adapt. Specialization may lead to the more efficient deployment of nurses, but it also increases the need for coordination. There is also the risk that their employability decreases due to a lack of flexibility. 

To conclude, parallelization may, in principle, increase the efficiency of nurses and doctors enormously and at the same time decrease the coordination efforts for their assigned work. At the system level, however, the coordination efforts increase, hence the need for slack. Therefore, it is not clear to what extent parallelization improves the efficiency and work characteristics of nurses and doctors. In practice, care providers need to monitor such systems during longer periods and continuously improve them. When demand uncertainty stays moderate over time, continuous improvement gradually leads to the more efficient utilization of nurses and doctors [20].

### 2.3. Systems of Tasks: Sequencing

Queuing system behavior not only applies to patient/client–doctor/nurse systems but also to the communication between healthcare professionals, especially when tasks are functionally defined and there is dependency between tasks. A nurse often has to wait until other nurses or doctors complete their tasks. The dependency of nurses on each other or others might be lowered by creating autonomous tasks (e.g., nurses with broad job descriptions responsible for all tasks but each responsible for a limited number of patients). This is a kind of horizontal coordination that contrasts with vertical or hierarchical coordination. An example of hierarchical coordination is when one nurse oversees the activities of a team of nurses and directs the nurses to where the work is. The need for coordination grows when task specialization and up- and de-skilling increase. De-skilling is a type of task shifting where tasks exchange from qualified workers to less-qualified workers [19].

The need for coordination is largely determined by the design of the healthcare system. The above-discussed systems were all one queue–one nurse/doctor systems, but what happens when patients have to go through a sequence of healthcare professions/organizations? See the figure below.

**Figure 3:**
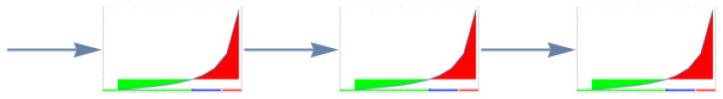
Multiphase healthcare system sequencing tasks leading to an open- loop system as uncontrollable queues develop. The green areas show the acceptable mean waiting times for patients. The red areas depicts unacceptable mean waiting times. The arrows represent patient flows. At the first workstation, both variable and uncertain inter-arrival and process times develop. When patients are finished at this first workstation, they enter the next queue, and this continues. The result is that the uncertainty and variety at the workstation are without control and are not communicated to the subsequent workstations. Without integrated control, the queues can even amplify. For the sequencing of tasks, care pathways need integrated control [21].

The example in Figure 3 shows a sequence of tasks with uncertainty in arrival times and service times resulting in long waiting times and high fluctuations for nurses and doctors, both at the station and system level. Such patient care pathways can be very attractive both for clinics and patients if it is possible to standardize the arrivals at the clinic, and if process times run in the same rhythm at each station. As an illustrative case, see the Gantt chart below showing a certain day of a highly standardized, multiphase office shift.

**Figure 4:**
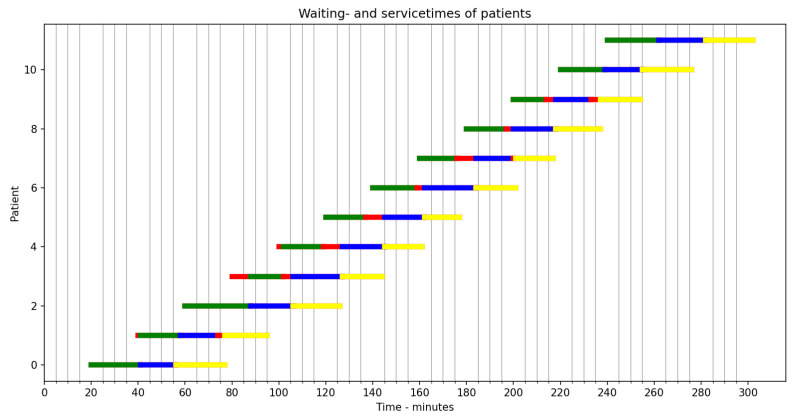
An example of a highly standardized three-phase outpatient clinic office shift. The color red represents waiting times for patients. The other colors represent processing times. The task time when entering the pathway is exactly 20 min. At each stage, the process time is 13 min plus 5 mean extension (Poisson distributed). This example shows the times of 12 patients. Calculating with this task time, the office hour is expected to last 280 min. It will sometimes be somewhat shorter and sometimes (considerably) longer.

Figure 4 shows that even if the office shift is very standardized, there is still a chance that more waiting time than anticipated is needed for the health professionals and patients involved. If the goal is to prevent excess time in this system, more standardization is needed, but this is not realistic, as the system is already highly standardized. Alternatively, fewer patients should be admitted, thereby increasing slack. Given the standard interval of arriving patients at the first stage, the bottleneck is the last station, as it takes the burden of the waiting and excess processing times of the upstream phases. To remedy this, the upstream phases should reduce their capacity or lower their occupancy rate (thus increasing their slack). The only alternative for reducing the capacity (and thus increasing slack) is by bringing in flexibility. For example, the doctor working in phase 1 could help with phase 2 and phase 3 when they are idle and patients are waiting downstream. This requires caregivers to be flexible and contribute to extra coordination. 

In [22] (p. 154), a distinction is made between paced and unpaced situations. In a paced situation, there is a strict upper limit to the task duration in each phase, while in an unpaced situation, this upper limit does not exist. The probability distribution of the actual task duration in paced systems is less skewed compared to unpaced systems [22] (p. 154). An important reason for the decreased skewness in the task duration in paced systems is that tasks are not completed or performed hastily. This is especially the case if tasks are assumed to be very standardized but in fact are not. 

Across various healthcare domains, we observe a tendency for systems to either strive for or assume standard timeframes for nursing tasks. The pressure of working within a paced system, even in situations where a more flexible, unpaced approach would be appropriate, or the pressure to work hastily can render the job unappealing. This strain may contribute to psychological stress, job dissatisfaction, and result in nurses either leaving the profession or experiencing adverse health effects. Developing a paced healthcare system is often carried out in combination with de-skilling (task shifting from qualified workers to less-qualified workers) [19]. The most famous example is probably the Aravind Eye Hospital in India [23]. Its healthcare system is the result of many years of experience of developing an integrated paced system with real-time coordination. The institute is renowned for developing and implementing evidence-based management methods.

The Aravind Eye Clinic started in 1975 with a clear mission: avoiding blindness. All patients needing treatment should receive it, including the very poor. The challenge to offer care to the very poor is that it is very expensive and time consuming, and there is also a lack of skilled staff. To realize its mission, the Aravind Eye Clinic chose to almost completely standardize all processes. The layout of buildings, workplaces, and equipment are adapted to these highly standardized processes. The standardized clinical pathways are also logical pathways which are monitored in real-time. As both surgeons and nurses are scarce, the practice of de-skilling was massively implemented. Specifically, ‘sisters’ (not registered nurses) perform specialized tasks, allowing surgeons to completely focus on their specialization. The productivity of surgeons at Aravind is substantially higher compared to other clinics [24]. Everything is organized to minimize the throughput time of patients in the hospital (from admission to discharge). The positions of patients and staff are always known in real-time. The scheduling system constantly matches patients, staff, and equipment. Due to the high degree of standardization, the system is almost completely paced with minimum slack time. It has now developed into the largest eye hospital in the world (in terms of number of patients served).

If paced systems can function as closed-loop systems, they may offer good working conditions for nurses and other healthcare workers but reduce the number of nurses compared to unpaced systems. Thus, paced systems may need less slack (see the example of the Aravind Eye Hospital). However, if the conditions for pacing are not satisfied, a healthcare system intended to be paced will be an open-loop system and have unattractive jobs and quality problems. This will have adverse effects on job satisfaction and stress, resulting in higher turnover and more absences due to sickness. In unpaced systems, slack is even more important compared to paced systems like that of the Aravind Eye Hospital. 

### 2.4. Systems of Tasks: Deliberate and Emergent Blocking

**Figure 5:**
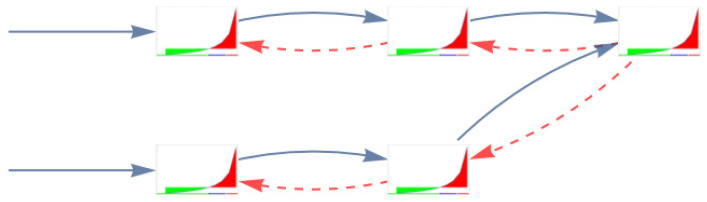
The spreading of blockages [17] (p. 81). The green areas show the acceptable mean waiting times for patients. The red areas depict unacceptable mean waiting times. The grey arrows represent patient flows. The red arrows blocking signals.

In healthcare, blocking is a common phenomenon: a department closes its beds as it is ‘full’, or blocking is induced because the next department of the clinical pathway ‘blocks’. If a department blocks not because other departments do so, then we call it deliberate blocking; if it blocks because other departments make it necessary to do so, then this can be referred to as emergent blocking (see also the text on ‘information entropy of networks’ in the Appendix A). Coordination beyond the simple queuing system is difficult, especially when different types of care (e.g., ICU and regular wards) are involved in different healthcare organizations (e.g., hospitals and home care). High occupancy rates in combination with a disregard for the level of variation will inevitably lead to blocking [16,25]. Figure 5 shows two patient streams. These streams may represent two completely different types of patients, involving different doctors and nurses, with one exception: the last resource—the ICU—is shared. A high access rate at the first station of one stream may, after some time, lead to the shared resource becoming highly occupied, subsequently blocking access for both streams. This is a systemic failure that causes a sequence of local blockages that run from down- to upstream (initially upstream; caused by one stream). For patients, quality issues arise, as this blocking requires staff to deal with types of patients that they may not be trained in dealing with or may not have been scheduled to deal with, as patients are ‘stuck’ in the ‘wrong bed’. In addition, the staff will have to handle a high level of fluctuation in their workloads. The overall ‘out-of-control’ nature of the system can leave staff feeling disoriented and overwhelmed. This sense of being ‘out-of-control’, rather than the average workload itself, can contribute to job dissatisfaction and potential health issues among nurses.

### 2.5. From Local to System Control

In advanced manufacturing, methods such as Lean, Lean Six Sigma, and TPS (Toyota Production System) are widely applied to improve efficiency and enhance overall quality. While techniques derived from these methods have found extensive applications in healthcare, there is no evidence that these methods have been deployed at the system level (of the care organization), according to a recent literature review [26]. A typical example of a commonly applied method by Lean companies is the system of production leveling, which involves carrying out the following steps: (a) Identify the minimal average mix of products ordered throughout the year. (b) Determine the time period for this product (this could be, for example, three to four weeks). For outpatient clinics, this means that when the type, mix, and volume of office hours have been determined, the average mix occurs every three to four weeks depending on the period chosen. (c) The staff scheduling, including staff rosters, should be aligned with this production cycle.

However, the real demand can be (somewhat) different for the current and next period, and as patient access times should be restricted, the deviation between the real and standard production mix should be absorbed. The chosen office hour system and the specialization degree of staff determines the difficulty of realizing this flexibility. If care organizations have decided to work as much as possible with standardized specialized office hours, there will be an enormous effort needed to adjust the standard production schedule, as many office hours have to be rescheduled, or staff have to be transferred to another office hour. In general, specialization can lead to much more control in specific office hours while leading to much more coordination at the system level and thus extra slack. This reasoning is the same for specialized doctors and nurses in the ward or those providing home care. 

The implications of this system extend to staff rostering, typically set for a fixed duration, such as six weeks. While these rosters aim to ensure the availability of healthcare staff and allow for personal (private) activities, once planned, nurses and doctors face limited flexibility to modify their schedules. However, in situations where production schedules lack stability, necessitating increased coordination and adaptability, or when staff absence arises due to illness, staff rosters become a source of frustration and uncertainty. In healthcare, the inherent combination of rigidity and instability renders rosters unattractive. The challenges associated with rosters contribute to a less appealing work environment, a significant factor that prompts nurses to either leave their current positions or seek employment in institutions with more attractive rostering systems. Above, we discussed paced and unpaced systems, and we argued that a paced system is introduced where the conditions for pacing are not fulfilled. The opposite may also be true, as a system that is unpaced but could be paced makes processes and working times much more predictable, creating more attractive jobs [27]. Organizations employing Lean methods at a systemic level invest years of dedicated efforts and persistent improvement to stabilize their manufacturing systems. However, as highlighted by Vijverberg et al. [26], hospitals seem to be lacking a comparable investment in attaining a similar level of control in their operations at the systemic level. It remains a topic of further research and experimentation in practice whether systemic-level stabilization is possible in complex healthcare environments. The Aravind Eye Clinic has demonstrated that it is possible, but that does not mean it is possible in all healthcare environments. 

## 3. Job and Organization Characteristics and Psychological Stress

### 3.1. Information Processing Theories of Coordination and Organizing

The absence of system control and slack results in a loss of professional autonomy, specifically the necessary autonomy required for nurses to work effectively [28]. From a cybernetic perspective, the organizational structure determines, to a large extent, the maximum quantity of information that can be processed and therefore the quantity and quality of coordination. The quality of coordination is, according to this theory, determined by the time needed to process the information [29]. Galbraith’s [30] cybernetic theory of organizations explains how coordination issues and a lack of perceived autonomy also result in problems at the system level. According to Galbraith’s theory, the creation of slack resources can be considered as a strategy to reduce the need for information processing among nurses (the cybernetic translation of ‘coordination’) and is inevitable. Organizations might operate without recognizing the existence of buffers and assume that they run very efficiently, but in fact, they do not know their own slack. If buffers are externalized, the cost of slack goes to others, such as patients experiencing treatment delays. When nurses consistently face stress in the completion of their tasks, they become the de facto buffers of the system. Without proper buffer management, the system can become inefficient, unstable, or externalize costs, making them the workers responsibility, causing burdens such as illness and financial strains on insurance companies due to increased sick leave.

The information theories of Shannon [31] and Galbraith [30] relate coordination to the required level of autonomy from an information processing view: If the demand of patients is uncertain, as well as arrivals at healthcare institutions, it is expected that the entropy rate of the patient departure process should either be equivalent or lower. The arrival of patients is usually assumed to follow a Poisson distribution [32]. At any phase in the service process, the already realized reduction in entropy should not reverse. An organization is a deliberately designed interdependence with boundaries. As Georgescu-Roegen [33] (pp. 213–214) notes, boundaries are needed for analyses both in time and in space. Also, to control the flow of resources going in and out of the organization, what is ‘our’ and what is ‘not-our’? However, boundaries in time and space are also needed, as our perception is limited [34] (p. 65). The possible length of the planning horizon is dependent on the extent that we can forecast, causing reductions in local entropy and lengthening the possible planning horizon, creating stability and cohesion [34] (p. 66). The degree to which a system or one of its parts is able to reduce the entropy is a measure of the autonomy. In other words, it is the level of independence of the environment. Thus planning can stabilize processes in healthcare institutions and should be promoted, but instability can be increased if plans do not capture the uncertainty of their environment, their institutions, and their departments (see also the example in Figure 4). 

### 3.2. Job Design and the Organization of Work

Claveranne and Pascal (2004) observe that the high variation in the workloads of nurses throughout the day is primarily caused by a lack of coordination among different staff groups within the healthcare organization [35] (p. 73). When nurses have periods of inactivity during the day, they find themselves waiting on others, and when they do work, they must compensate for lost time. The level of independence experienced by healthcare professionals is a crucial aspect of their work environment. The job of a healthcare professional can be characterized by the ‘strains’ between job decision latitude and job demands [36]. Research on optimal healthy job design has a long tradition, with different perspectives. Some researchers focus on preventing burnout [37,38], while others argue for job designs that prevent ‘high strain jobs’ or ‘low strain jobs’ [36,39]. Furthermore, Edmondson draws connections between workplace safety, particularly in healthcare settings, and psychological safety [40,41]. Despite varying contexts, studies have consistently pointed to healthy job characteristics that allow for autonomy, efficiency, and promote learning for both employees and employers. These characteristics include jobs with high demands, high decision latitude, and social support from leaders and team members. Such jobs, characterized by high demands and requiring autonomy with social support, create engaged employees, as described by the author of [42], cited by the authors of [38].

In addition to decision latitude, task standardization plays a crucial role in mitigating the level of task uncertainty required to navigate environmental uncertainties [43,44]. Environmental uncertainty can be ‘caused’ by other teams or departments. Task standardization is often accomplished by the standardization of skills through professional training [45]. While environmental uncertainty and task uncertainty are somewhat related, they are not entirely synonymous, as task standardization can serve as a buffer against environmental uncertainty. For example, Tummers et al. [44] showed that ICU nurses perceived less environmental uncertainty due to higher levels of task standardization compared to their counterparts in non-ICU settings. Task standardization has the potential to reduce the stress experienced by nurses, thereby increasing retention. Nevertheless, ill-designed task standardization can also heighten stress levels and compromise the quality of care, as demonstrated in our discussion of paced healthcare systems.

Studies on job characteristics and work stress among nurses have been repeated many times and in various countries in the last century and also in the aftermath of the COVID-19 crisis [46]. Gifford et al. [47] studied the required capabilities of hospitals to deal with uncertainty and analyzed how flexibility could be increased without sacrificing efficiency. The authors stress the need for hospitals to develop their “sensing and seizing capacities to be better prepared for and respond to environmental change” [47]. They recommend that hospitals should develop a long-term orientation by, for example, investing in forecasting capabilities and by better integrating their operations and technostructure. Having slack and using that slack in a collective manner is also essential [47]. The conclusions and recommendations are comparable to those of the information processing theory of organizations (developed by Galbraith [30]). A concrete translation of Galbraith theory to hospital settings was achieved by Winasti [29], building on the research of the authors of [48]. This study underscores the effectiveness of forecasting in hospitals, especially when complemented by staff flexibility through cross-training. Moreover, the study demonstrates that the impact for increasing the inflow and retention of nurses can be substantial even if the amount of flexibility is limited. 

### 3.3. Work Arrangements

The practical implementation of flexibility often diverges from the ideal. Gan [49] describes how, in response to nurse shortages and fluctuations in patient census, managers increasingly turn to alternative work arrangements. These work arrangements encompass shift work, temporary positions, and alternatives for permanent work. The impetus for this trend stems from both managerial necessity, requiring creative solutions, and nurses capitalizing on labor market scarcity to attain independence as self-employed healthcare professionals. The advantage of self-employment for nurses is their independence from collective wage agreements, allowing nurses to set their earnings based on what healthcare organizations are willing to pay. Self-employed nurses enjoy increased autonomy, deciding when and where they work, as well as the conditions under which they work. However, this shift towards more self-employed nurses can burden those employed directly by healthcare organizations, limiting their options and choices. Gan [49] observes that the temporary character of alternative work arrangements might increase the risk of developing transactional instead of relational interactions with colleagues and managers. Such a shift could potentially undermine autonomy and social support, key aspects of a healthy job design, as outlined in the Karasek model discussed above. There is a serious risk that, in the future, not only job characteristics will worsen, leading to reduced work quality, increased distress, burnout, and illness, but also work arrangements may decline, posing a significant threat to the recruitment and retention of nurses.

## 4. Discussion

### 4.1. Vicious Circles

While the term ‘healthcare systems’ is commonly used, the application of system theories is often overlooked. As we argued earlier, the absence of adequate slack time for nurses could lead to a concerning scenario with even fewer nurses in the future, which might in turn result in a lower capacity to care for patients. However, the true vicious circle arises when, due to the constrained nursing workforce, the already limited slack time is further diminished, rendering the nursing profession less attractive. This raises the question of whether we have sufficient knowledge about long-term developments regarding the nurse workforce.

The analysis above provides many new insights for the organization of processes but it has its limitations if we position it in the larger context of the entire healthcare system, the economy, and the educational system. The organization of processes within a healthcare organization (like any other organization) should be adapted somehow to the environment it operates in, and it should also have some independence. The balance between openness and independence is crucial. Healthcare organizations rely, to a large extent, on government policies that shape the education and training of healthcare professionals and determine healthcare budgets and immigration policies, to mention only a few of the policies affecting healthcare. Timeliness plays an important role in this context. The sustainability of healthcare organizations rely on a certain level of stability in these government policies. In this section, we address the following topics:

- Well-documented long-term perspectives that are often available in many countries but have little impact on policymaking.
- Policy making for short-term problems having long lasting effects.
- Immigration and how it has been used both as buffer (to avoid having a lack of staff) and as a way to reduce training costs. However, immigration patterns are changing.

The three above-mentioned topics are out of the control of individual healthcare providers, but they have major impacts impact on the healthcare sector. Two other topics in this discussion relate to the profession of nurses. Here, we see that individual healthcare organizations could have substantial influence. We also address the following two topics:

- Task shifting: Up- and down-skilling.
- Topics related to coordination responsibilities, as well as first- and second-order problem solving by nurses.

### 4.2. Short- and Long-Term Perspectives on the Nurse Workforce

Healthcare systems commonly employ scenarios and long-term forecasts to project the anticipated volume of doctors and nurses. For doctors, scenarios and forecasts are also made by specialization. These forecasts typically align with the Workforce Planning Framework Process, as outlined by Willis [50,51]. However, there is a scarcity of scenario models concerning the long-term requirements for the nurse workforce. A recent systematic literature review conducted by Davahli et al. [52] on system dynamics simulation studies related to workforces in the healthcare sector identified several relevant studies [53,54,55,56,57,58,59,60,61,62] but found that only one study was about nurses [61]. From this, we conclude that there seems to be a lack of long-term scenario studies about the nurse workforce. 

### 4.3. Short-Term Problem Solving—Long-Term Effects

The 2008–2013 economic crisis forced countries to take austerity measures and impose more constraints on healthcare budgets. Fleming et al. [4] describe how budget constraints led to restrictive and degrading working conditions for healthcare professionals. Policy changes also had an impact on a global level: for example, the migration of nurses was redirected from non-EU immigrants to EU immigrants in Western Europe [6]. Many countries froze nursing salaries and training funding. As a result of these measures, nurse shortages either emerged or intensified [6]. Short-term and long-term policies often contradict one another, or more precisely, many countries lack a cohesive long-term policy.

There are at least two explanations for why the salaries of nurses are frequently not competitive. One is that nursing salaries are subject to public sector decision making and limits to public sector budgets, leading public sector wages to generally lag behind. The other reason is that productivity growth in healthcare, like in the public sector in general, is lagging behind that of the private sector, which also limits opportunities for wage growth. The disparity between healthcare providers on the one hand and entities capable of boosting productivity (such as pharmaceutical companies and medical technology firms) on the other hand places healthcare providers at the lower end of the healthcare value chain. This dynamic allows pharmaceutical companies and medical technology firms to enhance productivity and claim a larger share of the overall value chain’s profits [63] (p. 2). This means that there will be salary increases in these companies through the introduction of new products on the healthcare market. At the same time, budget caps from the government and health insurance companies limit the room for salary growth for doctors and nurses. Winant’s observations [63] (p. 2) align with Baumol’s theory [64], which posits that service industries, like healthcare, have fewer opportunities for productivity gains and subsequent salary increases compared to manufacturing companies. Consequently, negative salary differentials with the manufacturing sector emerge. However, as these differences widen, corrections occur: individuals exit the healthcare sector and labor shortages necessitate wage increases, or unions take action. The result is an unstable nurse workforce.

### 4.4. Immigration

Higher-income countries have relatively more medical doctors and nurses who have been trained abroad [3] (p. 17). Migrant workers compensate for the lack of domestically trained health professionals. Countries like India and the Philippines, which are traditionally the countries where most nurses migrate from, have nursing schools specifically designed to train nurses for working abroad. The international recruitment and immigration of nurses are increasing, leading to nurse shortages in ‘sending’ countries like India [65]. The situation is, however, complex. In India, the number of nursing schools establishing training programs to help meet domestic healthcare needs has substantially increased during the last decade. At the same time, there are also big regional differences with nurses migrating within India. The incentives to work abroad include better wages and working environments [65]. Do these migration flows result in brain drain? Ortiga analyzed how, since the 1990’s, the Philippines actively promoted the education of nurses for working abroad and how it benefits from that through remittances and by ‘outsourcing’ the costs of training to the students [66]. Countries relying on immigrant nurses not only outsource the costs of training to the—often much poorer—‘sending’ countries but also to the nurses themselves. Following the economic crisis a decade ago, the Philippines experienced elevated unemployment rates among nurses who were trained to work abroad [66]. Nevertheless, as Buchan observed, transferring the risk of investment in training to the nurses themselves poses risks for the “receiving” countries as well [6]. During the economic crisis, a significant and variable change in nurse migration took place: nurses redirected their destination countries [6].

The employment of migrant nurses may be hampered by cultural and language-related problems. Nursing is a language-intensive occupation, and most countries set strict and high requirements regarding the language proficiencies of migrant healthcare workers. For this reason, migration between English-speaking countries has, in general, been more successful than between countries with different languages. A second impediment is that countries differ in the hierarchical structuring of the work. In some countries, nurses are granted more autonomy and decision-related latitude, while in others, nurses work under strict supervision and do not have much latitude in decision making. These cultural differences on the workplace often limit the successful integration of migrant nurses.

Immigration policies could be considered as a buffer strategy, where the costs of this buffer are for the potential immigrants or the sending country. Countries that can afford to use these types of immigration policies thus externalize the risks associated with training and recruitment of nurses. Nevertheless, this ‘buffering’ has risks for all involved parties. In contrast to the term itself, it amplifies the interconnectedness between healthcare and educational systems globally, rendering the global size of the nurse workforce more vulnerable. The situation regarding nurses in the aftermath of the global financial crisis in the Philippines demonstrates this.

### 4.5. Task Shifting: Up- and Down-Skilling

‘Down-skilling’ is applied when people with no nurse training perform tasks that used to be performed by nurses. Upskilling refers to situations where nurses must perform the tasks of, e.g., physicians. A Dutch study about the introduction of nurse practitioners showed the great variety between the types of healthcare institutions that work with nurse practitioners [67]. The main explanation is the lack of knowledge in healthcare organizations about the possibilities to transfer tasks from physicians to nurses (nurse practitioners). The same is probably true for de-skilling. A famous example of de-skilling on a large scale is the Aravind Eye Hospital in Madurai [24,68,69]. Years of standardization and process optimization to create an optimized closed-loop system made de-skilling possible [70,71].

While the specialization of nurses has the potential to enhance efficiency and raise the overall quality of care, it also introduces uncertainty about the composition of the workforce. This uncertainty, in turn, increases the risk of mismatches between supply and demand. The challenge of forecasting the required workforce becomes bigger, particularly in the long term, where aggregate planning—disregarding the specialization of nurses—is essential. As planning horizons shorten, plans should be more specific. Operational planning and scheduling require a detailed understanding of the exact requirements of nurses. Certain cross-skills are needed to enable a certain flexibility in assigning nurses to tasks (units, departments, or even healthcare organization). As there is a tension between specialization, cross-skills, and personal characteristics, the degree of flexibility needed is important. 

### 4.6. First- and Second-Order Problem Solving

Nurses also need time for problem solving. Edmondson makes a distinction between first- and second-order problem solving [72]. First order problem solving is ‘fixing’, i.e., solving problems often by work arounds. The underlying cause is not resolved, and the problems of other departments perhaps worsen. Referring to a 2003 study [73], Edmondson argues that nurses have no time for second-order problem solving. In fact, of all problem solving, only 7% is second-order problem solving. The lack of managing at the system level also seems to be a reason why healthcare organizations do not fully embrace system methodologies like Lean Six Sigma or the Toyota Production System [26]. Many barriers to solving problems related to working conditions remain in place. 

### 4.7. Limitations

An important limitation of our study is that it is mainly theoretical. Empirical model testing remains a topic for further research. The theoretical model allows for the quantification of the impact of an absence of slack on the discussed shortage and coordination problems. Moreover, while there are many good case studies that have applied queuing theory, the scope of these studies is often smaller and different from ours. We suggest that a research program should be developed via applying our approach in extended case studies, similarly to, for example, the referenced studies on the Aravind Eye Hospital.

Another limitation of our approach is that we ignored many other factors that might influence labor shortages, such as the public perception of the nursing profession, the wages of nurses, and individual preferences for work–life balance. Moreover, our analysis focuses on why nurses are ‘pushed out’ of the healthcare profession. We only paid limited attention to ‘pull factors’ that explain why jobs outside of the healthcare sector are more attractive. 

## 5. Conclusions

Using queuing theory and information processing theory, we analyzed factors that cause the work of nurses to become unattractive and also, to some extent, ineffective. The absence of slack causes stress and a perceived lack of autonomy on a personal level, while for the healthcare organization, a lack of slack diminishes the quantity and quality of the coordination mechanisms available to them.

The main conclusion has to be that there is no evidence that healthcare organizations seem to attempt to manage their organization as a system, including the explicit management of buffers, which is needed for closed-loop systems. A closed-loop system is a sine qua non for good working conditions for nurses. If no substantial policy changes aimed towards improving the management of healthcare based on the discussed system sciences are made, workforce problems will continue to exist and possibly worsen. Also, at the healthcare system level, forecasting, planning, managing, and the retention of nurses needs to be closely considered in relation to one another and other areas of healthcare policy and management [74].

Consciously creating slack is necessary at all system levels. A study by Baumann et al. offered an example of an intervention that explicitly creates slack [75], discussing the strategies used by the government of Ontario (Canada) and healthcare organizations to create slack, overcapacity, and improve the retention and professionalism of nurses. Baumann et al. evaluated the Nursing Graduate Guarantee (NGG) at one hospital organization. The NGG funds the hiring of nurses into temporary overcapacity (above normative staff) full-time contracts with extended orientation and mentorship [75]. Before the evaluation period, the nurses were primarily working part-time, leading to high turnover. After the introduction of the NGG, both the retention rate and professionalism increased substantially. This is an example of a program that needs extension.

In general, there is a need to develop national and international programs to organize professional healthcare work differently. The questions that need to be addressed are as follows:

- How can slack capacity be effectively created and used?
- What are ways to organize paced healthcare systems? See the Aravind Eye Hospital for an example.
- What are good models for task shifting?
- In what manner can the positioning of nurses within the governance structures of healthcare organizations be optimized to enhance their influential role in coordinating processes?

The current situation requires more than just incremental decision making or ‘muddling through’. Lindblom, in *The Science of “Muddling Through”* [76], explained the rationality of “muddling through”, which is a model that is politically more realistic and better fits human nature to find a good solution instead of an optimal solution. What appears to be an ad hoc process has, according to him, had an implicit rational course that, with the exchange of contradictions, leads to the best possible outcome. If a choice has to be made between ‘muddling through’ or a ‘rational model of analyzing all possible options’ and their outcomes and before making decisions, then risk management also plays a role: who will bear the risks if the outcomes are different than expected or hoped for? As we have seen, short term problems in healthcare workforce management take priority. The risks of this ‘muddling through’ are borne by nurses and, ultimately, patients.

We demonstrate the applicability of system theories often by ‘what goes wrong’. It can be argued that is easier to observe ‘decoherence’ than ‘coherence’. But ‘coherence’ can also be seen as an active process. As François Jullien writes, ‘Co-herence’ means ‘keeping together’ [77] (p. 77). In this line of thinking, introducing and adapting the approaches of systems such as Lean, Lean Six Sigma, and the Toyota Production System to the systems of the healthcare sector could work. 

The challenges are increasing. In many countries, there is a growing elderly population. The alternating periods of nurse oversupply followed by an excess demand for them no longer exist, and now there is an absolute scarcity of nurses. As a shortage of nurses is a characteristic of all aging societies, solving nurse shortages by immigration becomes less and less feasible. We also observe that immigration streams are redirecting. The options for improvements are running out. 

## Data Availability

Data are contained within the article.

## Appendix A. Queuing, Coordination, and Information Processing: A Summary of Theories and Their Relations

### Appendix A.1. Queuing

*Queuing system*: servers offering services to customers. If servers are occupied or not available, customers wait in one or more queues [78] (p. 118).

The *arrival process* is described by the mean *inter-arrival time*, the time between arriving customers, $\frac{1}{\lambda }$ per unit of time, for example 1 h. λ is the *arrival rate* per unit of time. The arrival times of customers are assumed to be independent of each other and stochastic [78] (p. 118).

*Service processes* are described by type and number of servers and their *processing times.* Servers can have their own queues or share one or more queues. Processing times are described by the mean processing time $\frac{1}{\mu }$ per unit of time. μ is the *processing rate* at a server. The processing times of customers are assumed to be independent of each other and stochastic [78] (p. 118).

A queuing system can have prioritization rules about the order of servicing customers, this is called the *queue discipline* of a queuing system [78] (p. 118). A queue discipline is a type of coordination.

It is important whether and how *pooling* is applied when designing queuing systems. Pooling is “the replacement of several ingredients by a functionally equivalent single ingredient” in a queuing system, and can be carried out in several ways: by “pooling queues (the demand), pooling tasks (the process) or pooling servers (the resources)” [14]. Pooling can influence the arrival patterns, the processing times, the utilization, and the coordination needs and capacity in a queuing system. When pooling increases, the coordination needs at the level of the queuing system increases, while the need to coordinate with other queuing systems decreases.

In queuing systems, we assume a relation between available *capacity* and the *service rate* μ. To control queuing, the arrival rate of customers, λ, should be less than the service rate; hence, λ<μ. In a single server model, the difference between λ and μ is an important indication of the efficiency of the system, as $\frac{\lambda }{\mu }$ captures the utilization degree of the server. The *degree of underutilization* $\frac{\mu -\lambda }{\mu }$ can not only indicate inefficiency but also the need to deal with fluctuations in the demand or time for coordination activities. We can distinguish between *stochastic demand* and *uncertain demand*. Stochastic demand means that demand is random and the random process is known. Uncertainty means that the process generating the demand is not known [20]. In healthcare, both can take place. An example of stochastic demand is the development of the COVID-19 pandemic. An example of uncertainty is the number of patients who will be visiting an open-office hour of a GP.

### Appendix A.2. Information Theory and the Coordination of Queuing Networks

The need for coordination and the time required for coordination can be made explicit by considering coordinators as queuing systems. Coordinators process information into decisions. An organization is therefore a network of two types of servers: production servers (e.g., diagnostics, treatment) and coordination servers (e.g., scheduling, staff assignment, self-management). Arrival patterns, process characteristics, available capacity, routings, etc., determine the need for coordination. All servers can have queuing in front of them. The quality of coordination depends on the actual and expected states of the servers and queues determining the amount of information to be processed. Uncertainty about the actual or expected states should be handled by *slack capacity*, by coordination, or both [29]. To determine the amount of information for coordination, and thus the amount of information processed by the coordination servers, Shannon’s concept of *information entropy* can be used [31]. This is defined as follows:

$$H\left(x\right)=-\sum_{i=1}^{n}P\left(x_{i}\right){log}_{2}\;P\left(x_{i}\right)$$

where *X* is a discrete random variable representing the average uncertainty about *n* outcomes (*x*_1_, *x*_2_, *…*, *x_n_*).

For a specific queuing system, the number of states, and thus the amount of information to be processed, can be limited. In a *network of queuing systems* (a hospital, a care network), the amount of required information processing depends on the level of global coordination. If the network is a completely *open-loop* system, each queuing system has its own local coordination and is not any on the network level, and the information processing is limited to the sum of the information entropies of the queuing systems. When the network becomes a completely *closed-loop* system, all queuing systems are locally coordinated, and all the relations between the queuing systems are coordinated on the network level. The information processing need is much higher compared to a network with only local coordination. The *information entropy of network* can be defined as follows [79]:

$$H_{graph}=-\sum_{i=1}^{n}\frac{deg\left(v_{i}\right)}{m}*{log}_{2}\frac{deg\left(v_{i}\right)}{m}$$

where *n* is the number of queuing systems, and *m* is the number of relations (edges) between the queuing systems. *deg*(*v*) is the degree of vertex *v* (the queuing system), the number of edges incident to *v.* Based on the above equation, it follows that if the number of vertices and degrees increase, the entropy, (the complexity of the coordination, increases exponentially. As total coordination is hardly possible in complex networks, cybernetic theories of organizations, like Galbraith’s organizational design theory [30], focus on complexity reduction (reducing the number of relations between queuing systems) while remaining sufficiently in control of the organization [80] (in our case, the healthcare organization).

### Appendix A.3. Probability Distributions and Information Theory

For simple systems, often, exponential distributions of waiting and inter-arrival times are observed. It is very likely that there are many intervals with short times and few intervals with very long times [81] (p. 340). The *exponential distribution* is defined as $p\left(x\right)=\lambda e^{-\lambda x},\;\;where\;\lambda \;is\;the\;mean\;value,\;and\;\lambda \;=\;\langle x\rangle .0\leq x\leq \infty$ [80] (p. 23). While the interval time distribution gives the time per event, the *Poisson distribution* gives the number of events per unit of time [81] (p. 340). The Poisson distribution is defined as $p\left(n\right)=\frac{\lambda^{n}e^{-\lambda }}{n!},\;n=0,1,2,\ldots ,N$ [80] (p. 23). Both distributions assume independent events.

If there would not be any order at all, the most reasonable assumption would be to assume a *uniform distribution* of inter-arrival times. Thus, if we know the expected mean time between arrival and when a new patient has just arrived, an estimation of when the next patient will come can be based on a uniform distribution. If, in some way, there are constraints (e.g., patients arrive after referral from GPs, midwifes, etc., who have office hours), then these constraints transform the uniform distribution into an *exponential distribution*, similar to a *Poisson distribution*. See the reasoning of Dill at al. [81] (p. 86) below.

Let us assume that at the beginning of each hour a dice with each side showing a number of patients arriving is thrown. The number of patients λ that arrive each hour is $\lambda_{min}\leq \lambda \leq \lambda_{max}$. Following the explanation of Dill et al. [81] (p. 86), the number of sides of the dice is the same as the amount of numbers of $\lambda_{min}\leq \lambda \leq \lambda_{max}$. “When the dice is fair for any number $\lambda_{i}$, the average score per roll $\langle \lambda \rangle$ is $\langle \lambda \rangle =\frac{\Lambda }{N}=\sum_{i=1}^{t}p_{i}\lambda_{i}$, where $\Lambda =\sum_{i=1}^{t}\lambda_{i}n_{i}$ and $n_{i}$ is the number of times face i showing up in N rolls” [81] (p. 86). The fair dice will predict a uniform distribution.

The above reasoning means that there is no sufficient reason not to assume a uniform probability distribution of the inter-arrival times of patients: having no information is the same as expecting a flat distribution [81] (p. 89). However, Barra et al. [82] argue that patients do arrive according to a Poisson distribution. Therefore, some form of organization has influence on the arrival patterns: constraints predict exponential distributions [81] (p. 89). The same reasoning applies to processing times within the healthcare organization.
